# Person-Specific Methods for Characterizing the Course and Temporal Dynamics of Concussion Symptomatology: A Pilot Study

**DOI:** 10.1038/s41598-019-57220-1

**Published:** 2020-01-27

**Authors:** Amanda R. Rabinowitz, Aaron J. Fisher

**Affiliations:** 1 Moss Rehabilitation Research Institute, Elkins Park, PA USA; 2 Department of Psychology, University of California Berkeley, Berkeley, CA USA

**Keywords:** Brain injuries, Neurological manifestations

## Abstract

Better characterization of acute concussion symptomatology is needed in order to advance clinical and scientific understanding of persistent concussion symptoms. This paper aims to illustrate a novel framework for conceptualizing, collecting, and analyzing concussion symptom data. To that end, we describe the temporal and structural dynamics of acute concussion symptoms at the individual-patient level. Ten recently concussion adolescents and young adults completed 20 days of ecological momentary assessment (EMA) of post-concussion symptoms. Follow-up assessments were completed at 3 months post-injury. Network modeling revealed marked heterogeneity across participants. In the overall sample, temporal patterns explained the most variance in *light sensitivity* (48%) and the least variance in vomiting (5%). About half of the participants had symptom networks that were sparse after controlling for temporal variation. The other individualized symptom networks were densely interconnected clusters of symptoms. Networks were highly idiosyncratic in nature, yet emotional symptoms (*nervousness*, *emotional*, *sadness*), cognitive symptoms (*mental fogginess, slowness)*, and symptoms of hyperacusis (*sensitivity to light, sensitivity to noise)* tended to cluster together across participants. Person-specific analytic techniques revealed a number of idiosyncratic features of post-concussion symptomatology. We propose applying this framework to future research to better understand individual differences in concussion recovery.

## Introduction

It is estimated that 15–30% of those who sustain a concussion go on to experience persistent symptoms^1^. Predicting who is at risk of poor recovery is a challenge, and evidence-based treatments are lacking. Although both neurogenic and psychogenic factors likely contribute, underlying causes of distress for a given patient may be difficult to uncover. By their very nature, concussion symptoms are non-specific and associated with non-injury factors including post-traumatic stress^2^, depression and anxiety^3^, and other premorbid risk factors^4–6^. There is a growing appreciation that post-concussion symptomatology is best conceptualized according to a bio-psychosocial model—wherein evolving pathophysiological processes interact with pre-injury vulnerabilities, environmental stressors, and psychological factors to produce symptoms. The underlying pathophysiology is itself complex, unfolding across the levels of cellular changes, brain network disruption, and perturbation of patients’ experiences and social environments^7,8^. Heterogeneity at each of these levels leads to symptom profiles that are both complex and idiosyncratic.

The multifactorial and individual nature of post-concussion symptoms is not well captured by a *latent disease model*, which assumes that symptoms arise as the result of a singular pathological process—in the case of concussion, presumably a neurometabolic cascade of events triggered by transient deformation of brain tissue in response to trauma. This assumption underlies the common practice of using the sum total of symptom-checklist items as an index of severity, which treats symptoms as though they (i) represent the same underlying process across individuals, (ii) are interchangeable indicators of injury severity, and (iii) are relatively stable over time. A recent review by Iverson (2019) argues that a latent disease or *common cause* theory is particularly inappropriate in the case of *prolonged* symptoms—or symptoms that persist beyond the typical recovery window over which neurometabolic changes in the brain return to baseline—and proposes adopting a network model of concussion symptoms instead^7^.

Network modeling approaches have been applied to complex systems of inter-related phenomena, including brain connectivity, social relationships, and more recently, symptoms and behavioral processes underling medical and psychiatric disorders. Network models consist of nodes, representing measured constructs, and edges, reflecting covariation between them. In symptom network models, rather than reflecting white matter pathways or social bonds, edges may represent a shared pathophysiologic process^9^ (e.g. fear conditioning accounting for covariation between fear and avoidance). In psychopathology, network theory stands in contrast to the latent variable and disease models, which purport that disorders arise from single underlying disease entities. Instead, networks are complex, with causality distributed across the self-reinforcing connections among nodes. This approach is particularly appropriate for syndromes, like post-concussion syndrome^7^, comprised of inter-related symptoms that are mutually reinforcing. Time series data collected from a single patient can be subject to network modeling, allowing characterization of the dynamic inter-relationships among symptoms on the individual-patient level.

Applying person-specific analytic techniques and network theory to concussion symptoms may better characterize individuals in the acute-injury phase. The current conceptualization of post-concussion symptoms implies a latent disease model, wherein symptoms arise as the result of a singular pathological process—presumably transient neurometabolic disruption caused by mechanical deformation of brain tissue. However, not all post-concussion symptoms track with the temporal course of neurometabolic recovery, suggesting that some symptoms may be caused or maintained by other biological (e.g. autonomic dysfunction, diffuse axonal injury) or psychosocial (e.g. stress, avoidance) processes. Network modeling has been proposed as an approach for characterizing “hybrid models” of disease—conceptual models that describe symptoms arising from a common cause (e.g. brain trauma) that are then maintained by interrelationships between symptoms. Hybrid models have been evoked to describe post-traumatic stress disorder, the onset of which is due to exposure to psychological trauma, while its maintenance is fueled by direct interactions between symptoms (e.g., anger resulting in ongoing difficulty concentrating)^10^. In a similar fashion, brain trauma may serve as a root cause of concussion symptomatology that is maintained as a result of mutually reinforcing links between symptoms.

Traditional research designs that collect data at one, or few, occasions within a large sample cannot uncover potential heterogeneity in the temporal or structural dynamics of acute post-concussion symptoms. Rather, a *person-specific* approach is needed to characterize idiosyncratic patterns of covariation among symptoms. In clinical contexts the goal is to make inferences about processes that occur *within the individual;* yet, aggregate-level research findings seldom hold at the level of the individual subject. In fact, an analysis of data from six sites in the United States and the Netherlands recently demonstrated that the patterns of variation and covariation within-subjects do not align with those between-subjects physiological processes^11–16^. Methodologies that characterize symptom variation in *individuals* across *time* are needed to optimize treatment outcomes for individual patients. Thus, the impetus exists to pursue the measurement and analysis of personalized time-series concussion symptom data, now readily realizable with smartphone “apps” for delivering ecological momentary assessments (EMAs).

Person-specific time series analysis provides additional advantages for specifying temporal dynamics. To date, network models of longitudinal or time series data have largely assumed that the mean levels of network nodes and the covariation among nodes is consistent across time – a condition known as stationarity. This is an untenable assumption for concussion symptoms. As time progresses from the moment of injury, individuals are likely to recover along a linear or curvilinear trajectory. In addition, some symptoms may be influenced by diurnal variations in arousal and activity. Failing to account for these temporal sources of variation could obscure symptom topography (c.f.^17^). This paper represents the first instance of time series network models that include longitudinal trends and diurnal cycles within the estimation of the network.

The present study explores the idiographic structure of concussion symptomatology in a sample of recently concussed adolescents and young adults. We aim to illustrate a framework for conceptualizing, collecting, and analyzing concussion symptom data that accounts for potential nonstationarity—systematic changes in symptom mean levels over time. We describe the temporal and structural dynamics of acute concussion symptoms at the individual-patient level, and demonstrate how to accommodate symptom recovery in time series-based network models.

## Method

This study was conducted in compliance with APA ethical standards in the treatment of our human subjects and was approved by the institutional review board of the Albert Einstein Healthcare Network. Each participant, or their parent for individuals 17 years of age or younger, provided written informed consent. Participants who were minors (under 18 years old) were informed of all study procedures and provided assent.

### Participants

10 recently concussed adolescents and young adults were recruited from an outpatient concussion clinic. Participants were required to meet the following inclusion criteria: a) diagnosis of concussion by physician, b) 15–35 years of age, c) fluent in English, d) Post-concussion Symptom Scale (PCSS) score > 0 at the time of enrollment, d) documented PCSS score >  = 10 between the time of injury and enrollment, e) injury occurred within 30 days of enrollment. Potential participants were excluded if they had loss of consciousness > 30 minutes or post-traumatic amnesia > 24 hours (indicative of a more severe brain injury), or if they had previous history of moderate to severe traumatic brain injury or neurological disorder. Prior concussion was not grounds for exclusion. Participants aged 18 and over underwent an informed consent procedure. For children 15 to 17 years old, a parent or legal guardian provided consent on behalf of the participant, and the participant underwent an assent process.

### Procedure

The EMA protocol was designed using the LifeData^R^ platform. A LifePak was developed based on the PCSS, which has respondents rate the extent to which they are experiencing 22 concussion symptoms on a Likert scale ranging from 0 “not at all” to 6 “extremely.” Participants received notifications prompting them to complete the PCSS 5 times per day for a period of 20 days. Notifications were delivered at 3-hour intervals timed for “morning,” “early afternoon,” “late afternoon,” “evening,” and “night” according to three preset schedules. After a notification was received the participant had 90 minutes to respond before the assessment window expired. Each participant was given their choice of assessment schedules based on which best matched their waking hours.

Upon enrollment, a research assistant downloaded and trained participants in using the RealLifeExp app. All participants were given the option of using a study-provided iPod touch or their personal device. A neuropsychological battery was administered at the acute visit and repeated at 3-months post injury (described in Supplemental Material).

### Approach to data analysis

EMA data were analyzed via two separate, parallel approaches intended to account for nonstationary processes in the data. In the first of these, nonstationarity was handled by including temporal variables in the estimation of each partial correlation network. By including temporal variables in the estimation process, sources of nonstationarity are effectively partialed out of the pairwise relationship between any two symptom nodes in the network. Additionally, edges between symptom nodes and temporal variables reflect significant temporal variation in the respective symptom. Linear, quadratic, and cubic trends were included to account for potential symptom recovery (or exacerbation) during the measurement period and 12-hr and 24-hr sinusoidal variables were included to account for possible diurnal and ultradian variation. Twenty-four-hour and 12-hour cycles were estimated consistent with methods provided by Flury and Levri^18^, which require a sine and cosine term for each frequency. All network models were estimated in the R package qgraph^19^.

The second approach comprised three steps for identifying and removing temporal processes from symptom variation in order to model the residuals. First, a least absolute shrinkage and selection operator (LASSO)-regularized regression model was used to determine the presence of temporal variation in each symptom, for each person, person by person. LASSO regression produces sparse models due to L_1_ penalization. In addition to protecting against overfitting, this feature is useful for variable selection—coefficients shrunk to zero are omitted from the model. Next, an ordinary least squares regression model with the retained temporal variables was run for each symptom. The residuals from these models were operationalized as the stationary variation in each symptom and were employed in the estimation of contemporaneous networks, otherwise consistent with the models generated in the first approach. Thus, the second approach yielded a partial correlation network of residualized symptoms with all variance attributable to trends and cycles removed prior to estimation.

## Results

The sample included 5 men and 5 women. Average age was 20.4 years (SD = 6.8). Most participants sustained their injuries as a result of sports, one in a fall, and two in vehicular accidents. Two of the ten participants reported loss of consciousness (LOC < 30 minutes) and post-traumatic amnesia (PTA < 6 hours). In the other 8 cases, participants reported alteration of consciousness, but no LOC or PTA. Nine participants were involved in sports at the time of their injuries. Average time to return to competitive play was 35 days (SD = 21.3). Eight participants were full-time students, and average time to return to full academic activities without accommodations was 20 days (SD = 11.7). Neuropsychological data is reported in Supplementary Table 1. The average EMA response rate was 73% (range from 49% to 95%). Six of the 10 responded to at least 80% of prompts.

### Conditioning on time within network models

The first approach to handling nonstationary variation in concussion symptoms involved conditioning nonstationary symptom variation on time by including temporal variables within each individual’s contemporaneous network model. Variables for linear, quadratic, and cubic trends, and 24-hr and 12-hr cycles were included during model estimation in order to partial out nonstationary effects. Additionally, edges between symptoms and temporal variables reveal specific temporal patterns in symptom variation. All ten network models are available on the open science framework at https://osf.io/9sqz5/.

For each individual, we estimated a sparse partial correlation network using LASSO regularization, implemented in R (version 3.3.1; R Core Team, 2016) with package qgraph^20^. As noted above, temporal variables were embedded as covariates. Thus, edges between concussion-symptom nodes and temporal nodes reflect trends or cycles in the given symptom. It should be noted that not all patients experienced all of the measured concussion symptoms. Network models therefore varied in the number of included variables.

### Network models with detrended residuals

The second approach to accounting for nonstationarity in concussion symptoms required us to conduct a set of regression analyses for each symptom, for each individual, in order to return model residuals conditioned on time. These residuals were then used for the estimation of stationary network models.

### Regression models

LASSO regression models were used to select appropriate temporal variables for detrending. After variable selection, an ordinary least squares regression was used to remove temporal variation. Residual data were then used to estimate contemporaneous network models. Table 1 reports the model R^2^ for each symptom for each individual, reflecting the degree to which symptom variation was driven by trends or cycles. Of note, symptom endorsement was not uniform across patients. One individual—patient five—endorsed only two symptoms, headaches and fatigue. Among those symptoms endorsed by participants, regression models explained an average of 25% of the variance, with a range from 0.00 to 82%. The variables best-explained by temporal variation were *light sensitivity* (*mean* = 48%), *noise sensitivity* (*mean* = 38%), *feeling foggy* (*mean* = 32%).

**Table 1:** Percent of variance (R^2^) in individual concussion symptoms accounted for by temporal variation (linear, quadratic, and cubic trends; 24-hr and 12-hr cycles).

	P01	P02	P03	P04	P05	P06	P07	P08	P09	P10	Avg.
Headache	0.13	0.09	0.00	0.00	0.47	0.00	0.26	0.32	0.60	0.26	**0.21**
Nausea	0.12	0.02	0.06	0.32	—	0.18	—	—	—	0.00	**0.12**
Vomiting	—	—	—	0.10	—	0.00	—	—	—	—	**0.05**
Dizziness	0.15	0.00	0.00	0.11	—	0.00	0.37	0.20	—	0.00	**0.10**
Fatigue	0.32	0.08	0.27	0.14	0.28	0.10	0.15	0.23	0.55	0.15	**0.23**
Drowsiness	0.00	0.00	0.10	0.17	—	0.13	0.42	0.05	0.32	0.00	**0.13**
Light	0.52	—	0.57	0.10	—	0.47	—	—	0.79	0.40	**0.48**
Noise	0.63	—	0.47	0.06	—	0.53	—	—	0.56	0.00	**0.38**
Irritability	0.65	0.00	0.32	0.24	—	0.41	0.00	0.30	—	0.13	**0.26**
Sadness	0.16	0.00	0.11	0.50	—	0.19	—	0.03	—	0.08	**0.15**
Nervousness	—	0.00	—	0.41	—	0.49	—	0.20	—	0.13	**0.25**
Emotional	0.59	0.00	0.06	0.33	—	0.22	—	0.00	—	0.26	**0.21**
Numb, Ting.	—	—	—	0.27	—	—	—		—	0.00	**0.14**
Slow	0.69	—	0.00	0.17	—	0.00	0.40	0.00	0.72	0.00	**0.25**
Foggy	0.38	0.47	—	0.20	—	0.14	0.51	0.00	0.82	0.00	**0.32**
Diff. Concen.	0.47	0.05	0.59	0.23	—	0.31	0.40	0.00	0.15	0.00	**0.24**
Memory	0.00	—	—	0.14	—	0.38	0.44	0.15	—	0.00	**0.19**
Vision	0.00	—	—	0.48	—	—	—	—	—	—	**0.24**
Avg.	**0.32**	**0.06**	**0.21**	**0.22**	**0.38**	**0.22**	**0.33**	**0.12**	**0.56**	**0.09**	**0.25**

Note: P01–P10 = participants 1–10; The en-dash symbol (−) = insufficient variance/symptom not endorsed by individual; 0.00 = no predictors retained in LASSO regression model.

**Figure 1:**
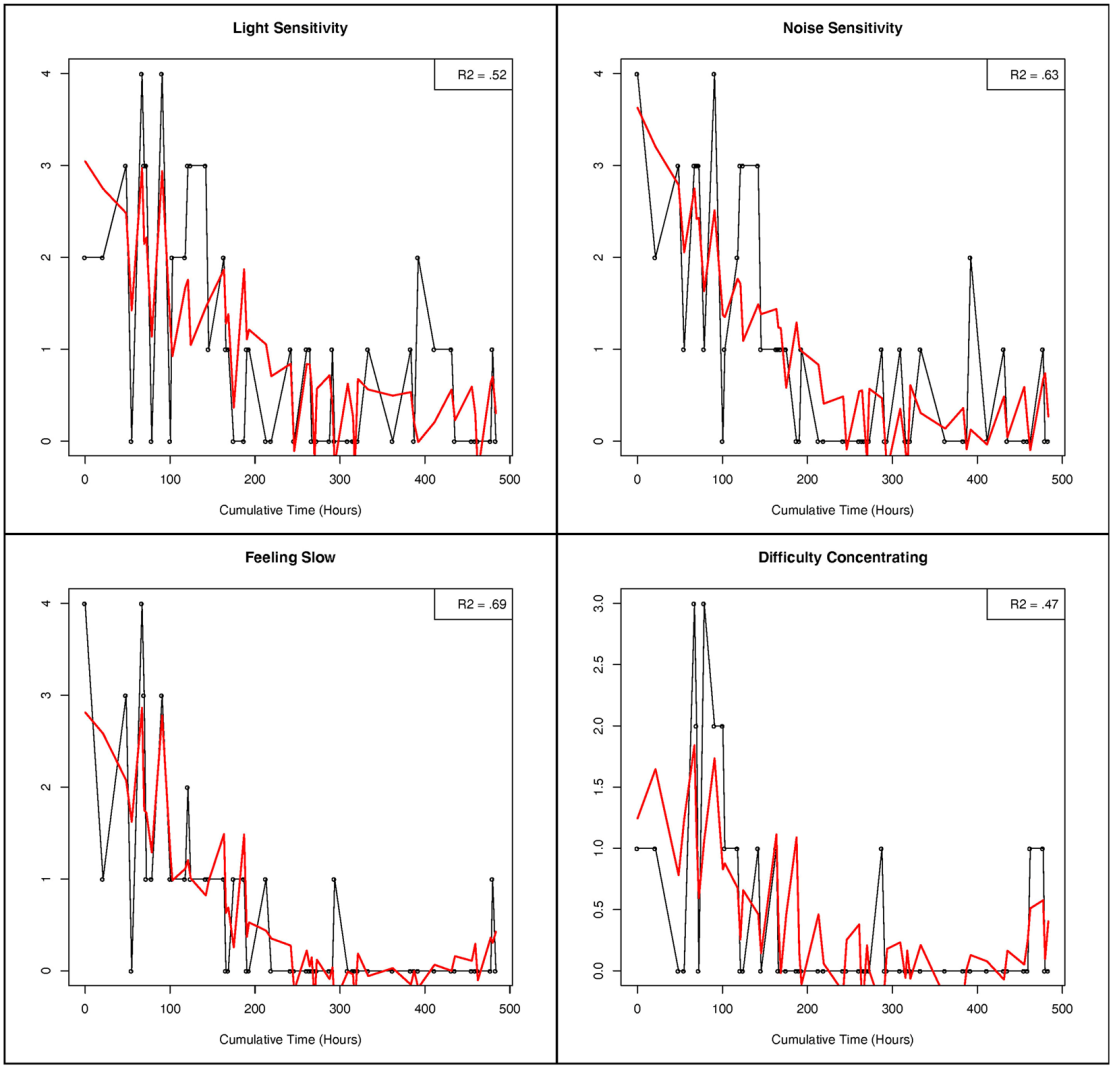
Contemporaneous (idiographic) networks for Patient 1 (top row), Patient 6 (middle row), and Patient 7 (bottom row). Detrended symptom networks are on the right hand side.

The regression models for each symptom, for each patient, are available on the Open Science Framework at https://osf.io/9sqz5/. Figure 1 presents raw data and predicted values for *light sensitivity*, *noise sensitivity*, *feeling slow*, and *difficulty concentrating* for Patient 1.

### Network models

Detrended symptom data were used to estimate a sparse partial correlation network using LASSO regularization, once again implemented in qgraph. All ten network models are available on the open science framework at https://osf.io/9sqz5/.

**Figure 2:**
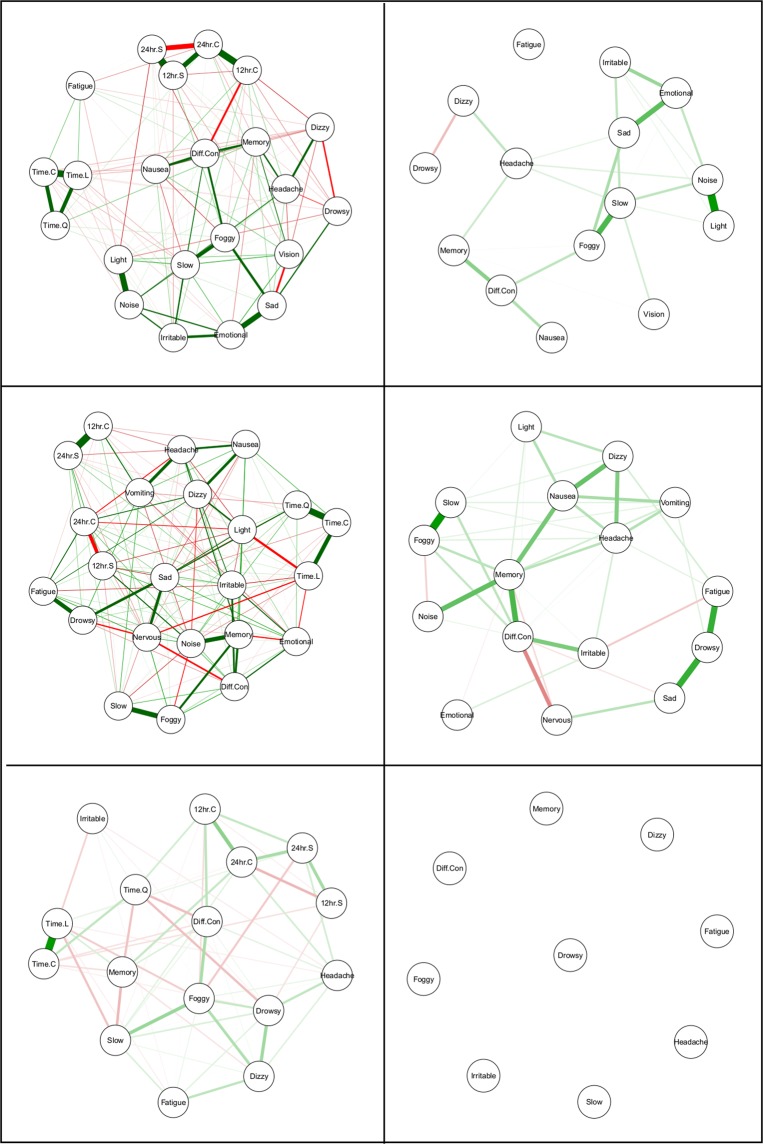
Raw data, predicted values, and variance accounted for in light sensitivity, noise sensitivity, feeling slow, and difficulty concentrating for Patient 1.

Figure 2 presents the contemporaneous partial correlation networks for Patients 1, 6, and 7 (top, middle, and bottom row, respectively). Networks with embedded temporal variables are on the left and networks with detrended symptom variables are on the right.

### Individual patient results

#### Patient 1

As reported in Table 1, temporal variables accounted for an average of 32% of the variance in symptom variation during the measurement period for Patient 1. Whereas, trends and cycles accounted for zero variance in memory and visual problems, temporal variation accounted for 47%, 52%, 63%, 65%, and 69% of the variance in *difficulty concentrating*, *light sensitivity*, *noise sensitivity*, *irritability*, and *feeling slow*, respectively. All five of these variables exhibited linear, quadratic, and cubic trends, as well as 24-hr and 12-hr cycles.

Consistent with symptom-level regression models, the most central symptom node in the raw data network model for Patient 1 was the 24-hr cosine term, with the 24-hr sine term the third-most central node. In all, temporal nodes were six of the eight most central nodes in the model, with *feeling slow* (second-highest) and *sadness* (sixth-highest) the other most central nodes. Examining the network model with detrended symptom nodes, *noise sensitivity*, *feeling slow*, *feeling foggy*, *emotional*, and *light sensitivity* were the five most central nodes.

#### Patient 2

Temporal variables accounted for an average of only 6% of the variance in symptom variation for Patient 2, with *feeling foggy* an outlier (R^2^ = 47%). Feelings of fogginess exhibited linear, quadratic, and cubic trends, as well as 24-hr and 12-hr cycles.

The raw data network model reflected this lack of temporal variation, with temporal variables comprising the five lowest centrality values and seven of the lowest eight. The most central symptoms were *difficulty concentrating*, *nervousness*, *dizziness*, *irritability*, and *emotional*. In the detrended network, *difficulty concentrating*, *nervousness*, and *dizziness* were again the three most central, with *sadness* and *headaches* the fourth and fifth most central.

#### Patient 3

Temporal variables accounted for an average of 21% of the variance in symptom variation for Patient 3. Temporal variables accounted for 32%, 47%, 57%, and 59% of *irritability*, *noise sensitivity*, *light sensitivity*, and *difficulty concentrating*, respectively. All four of these variables exhibited linear, quadratic, and cubic trends, as well as 24-hr and 12-hr cycles.

Wave-like temporal variation appeared to be the most influential force in the raw data network. That is, the *cubic* trend node was the most influential node in the network, followed by 24-hr and 12-hr cycles. Thus, this patient’s symptoms were marked by rising and falling patterns at ultradian, diurnal, and weeks-long frequencies. *Emotional* and *noise sensitivity* were the most central symptom nodes in the raw data network. Only four variables exhibited any strength centrality in the detrended network. These were *emotional*, *sadness*, *drowsiness*, and *fatigue*.

#### Patient 4

Temporal variables accounted for an average of 22% of the variance in symptom variation for Patient 4. Of note, Patient 4 was the only patient who endorsed all symptom variables. Regression models returned temporal predictors for all symptoms except for *headaches*. Temporal variables accounted for 41%, 48%, and 50% of the variance in *nervousness*, *vision problems*, and *sadness*, respectively. All three of these variables exhibited linear, quadratic, and cubic trends, as well as 24-hr and 12-hr cycles.

Nodes for 24-hr cyclic variation and a linear trend were the most central and third-most central nodes respectively. Symptom nodes for *drowsiness*, *sadness*, *feeling slow*, and *feeling foggy* were the second, fourth, fifth, and sixth most influential. The detrended network exhibited consistent results, with *feeling foggy*, *feeling slow*, *drowsiness*, *fatigue*, *emotional*, and *sadness* the six most central nodes in the network.

#### Patient 5

Patient 5 only endorsed two symptoms during the measurement period, *headaches* and *fatigue*. Temporal variation explained 47% and 28% of the variance in these variables, respectively; and both exhibited linear, quadratic, and cubic trends, as well as 24-hr and 12-hr cycles.

Regarding the raw data network model, temporal variables were the most central nodes, with the two symptom nodes exhibiting the least and second-least centrality. Twenty-four-hour cyclicity was the most influential of the temporal nodes. Given that this patient endorsed only two variables, the centrality of the detrended network cannot be interpreted, as both nodes exhibit an equivalent number and strength of connections.

#### Patient 6

Temporal variables accounted for an average of 22% of the variance in symptom variation for Patient 6. Temporal variables accounted for 41%, 47%, 49%, and 53% of *irritability*, *light sensitivity*, *nervousness*, and *noise sensitivity*, respectively. All four of these variables exhibited linear, quadratic, and cubic trends, as well as 24-hr and 12-hr cycles.

Despite the influence of temporal variation in the regression models, the most influential temporal node—24-hr cyclic variation—was only the seventh most central node in the raw network. However, as depicted in Fig. 2, Patient 6 exhibited a densely interconnected network, with relatively high strength centrality for *all* nodes in the network. The most central symptom nodes were *noise sensitivity*, *memory problems*, *nervousness*, *sadness*, and *headaches*, respectively.

Interestingly, the removal of temporal variation from symptom fluctuations during detrending had a marked effect on the centrality of the detrended network. The most central node from the raw network, *noise sensitivity*, was now the third least central node. The most central nodes in the detrended network were *memory problems*, *difficulty concentrating*, *nausea*, *feeling foggy*, and *headaches*.

#### Patient 7

Patient 7 endorsed nine of the 18 symptoms, with all but *irritability* explained to some degree by temporal variation. Altogether, temporal variation accounted for an average of 33% of the variance in symptom variation, explaining 40%, 40%, 42%, 44%, and 51% of the variance in *feeling slow*, *difficulty concentrating*, *drowsiness*, *memory problems*, and *feeling foggy*, respectively. Of these, *drowsiness*, *memory problems*, and *feeling foggy* exhibited linear, quadratic, and cubic trends, as well as 24-hr and 12-hr cycles. *Feeling slow* exhibited linear and quadratic recovery, and *difficulty concentrating* exhibited linear, quadratic, and cubic recovery trends, and a 12-hr cycle.

Given the influence of recovery in the regression models, it is unsurprising that linear time was the most influential node in the raw data network. The most influential symptoms were *feeling foggy* (second), *drowsiness* (fourth), *feeling slow* (sixth), and *difficulty concentrating* (seventh). Underscoring the importance of temporal variation for this patient, the detrended symptom data returned an empty network model, indicating no substantive covariation between symptoms. Thus, a plausible conclusion is that symptom covariance was largely, or even exclusively dependent on recovery trajectories and daily cycles.

#### Patient 8

Temporal variables accounted for an average of only 12% of the variance in symptom variation for Patient 8, with a range of 0% to 32%. Nevertheless, a 24-hr cycle and linear recovery trend were the most central nodes in the raw symptom network and only two variables exhibited any strength centrality after detrending (*nervousness* and *emotional*).

#### Patient 9

Although temporal variables accounted for an average of 56% of the variance in symptom variation during the measurement period for Patient 9, these results may be inflated. Patient 9 exhibited a steep, early recovery trajectory. Thus, linear and quadratic terms accounted for a significant portion of variation in all but one of the eight endorsed symptoms, with the exception being *difficulty concentrating* (R^2^ = 15%).

Consistent with the aforementioned recovery trajectory, quadratic time was the most central node in the raw symptom network. When examining expected influence—the degree to which changes in a given node would be expected to have downstream influence on other nodes (Robinaugh, Millner, & McNally, 2016)—quadratic time dwarfed all other nodes, exhibiting at least twice as expected influence as any other node in the network. *Noise sensitivity* (second), *feeling foggy* (fourth), and *feeling slow* (fifth) were the most central symptom nodes in the raw symptom network. The three most central nodes in the detrended network were *feeling slow*, *headaches*, and *noise sensitivity*.

#### Patient 10

Temporal variables accounted for an average of only 9% of the variance in symptom variation for Patient 10, with *light sensitivity* an outlier (R^2^ = 40%). *Light sensitivity* exhibited linear, quadratic, and cubic trends, as well as 24-hr and 12-hr cycles. This symptom notwithstanding, regression models failed to account for any variance in nine out of 16 symptom variables endorsed by Patient 10.

Despite the paucity of temporal influence at a symptom-by-symptom level, the most central nodes in the raw data network were the sine and cosine terms for a 24-hour cycle. In both raw and detrended networks, the three most central symptom nodes were *feeling foggy*, *feeling slow*, and *sadness*.

## Discussion

We illustrate an application of EMA and network theory to the study of post-concussion symptomatology, with the hope of stimulating research bringing these methods to bear to answer unresolved questions about concussion recovery. Importantly, we apply these methods within a *person-specific* analytic framework allowing examination of symptom dynamics on a person-by-person basis. Results revealed a number of idiosyncratic features of post-concussion symptomatology, with both temporal and structural dynamics differing across individuals.

Temporal contributions to overall symptom severity ranged widely across *symptoms* and across *people* within our sample. In the overall sample, temporal patterns explained the most variance in *light sensitivity* (48%) and the least variance in vomiting (5%). Understanding the temporal dynamics that influence specific symptoms may have implications for identifying the underlying mechanisms driving those symptoms *within* and *between* individuals. Individual symptoms that show linear/curvilinear trends may be indicators of neurometabolic recovery, whereas those that show sinusoidal patterns may reflect diurnal processes—e.g. sleep/wake cycle or diurnal metabolic rhythms. Although the time-dependency of symptoms varied across participants, some symptoms were more consistently associated with temporal trends than others—for example, temporal trends accounted for more than 30% of the variance in *nausea* for 1 of 6 participants reporting the symptom, as compared to 5 of 6 participants reporting *light sensitivity*. This information could help clinicians prioritize certain symptoms in their diagnostic and prognostic decision making. For example, ongoing neurometabolic disturbance may be indicated by persistence of symptoms that typically follow an acute recovery curve.

At the patient level, symptom networks with relatively weak temporal influence may indicate the presence of other factors contributing to symptom severity. For example, Patient 2 exhibited a symptom network that was weakly influenced by temporal factors, and highly influenced by emotional symptoms (*nervousness, irritability, emotional)*. For this patient, relatively stable psychosocial processes may be driving or maintaining concussion symptoms. Other aspects of symptom network structure could point to highly influential factors that may be advantageous targets for intervention. Both Patient 2 and Patient 6 reported cognitive symptoms, however; in Patient 2’s network, cognitive symptoms were peripheral to headache, but in Patient 6’s network they were more central. For Patient 9, *feeling slow*, *headaches*, and *noise sensitivity* were the most influential symptom networks in the detrended data, suggesting that treating these symptoms may lead to overall improvement.

The present study provided two methods for identifying and accounting for nonstationary variation in concussion symptoms. As time series approaches become more prevalent in clinical research areas, methods such as these will be vital—given the changes in mean level inherent in recovery and treatment-response profiles, clinical researchers need to be able to investigate variables of interest without the confounding influence of nonstationarity. Identifying and conditioning on sources of nonstationarity converts these influences from confounds—or sources of noise—into meaningful signals that can be partialed out of statistical relationships and targeted for clinical insights in their own right. Both of the approaches in the current study modeled the partial correlations between symptom nodes after controlling for temporal variation; however, in the first approach, temporal variation was partialed out of the pairwise relationships between symptoms, and in the second, temporal variation was partialed out of each symptom, independently, before symptom associations were modeled in network models. Each approach confers potential diagnostic and heuristic advantages. Whereas, embedding temporal variables within network models can provide visual identification of temporal patterns in concussion symptoms (via pairwise edges between symptoms and temporal variables), network modeling of detrended variables provides much sparser models that may better-illuminate symptom to symptom relationships.

The detrended networks revealed that some individual’s symptom networks were sparse after removing temporal variation, suggesting that most of the variance in symptoms was due to time, rather than mutually reinforcing relationships between symptoms. The edges that remained in these networks, for the most part, connected symptoms with phenomenological overlap (e.g. *sadness* and *emotional*; *fatigue* and *drowsy*), and in a few cases, weaker associations suggestive of potential causal links between distinct symptom clusters (e.g. the link between a *sadness-emotional* cluster and a *fatigue-drowsy* cluster in Patient 3).

Roughly half of the sample had more densely connected networks characterized by interconnected clusters of symptoms. Symptom clusters may indicate manifestations of a common underlying process, whereas the paths connecting clusters could reflect mutually reinforcing links between processes. Symptom networks were largely idiosyncratic, however, some commonalities across patients emerged. Emotional symptoms (*nervousness*, *emotional*, *sadness*), cognitive symptoms (*mental fogginess, slowness)*, and symptoms of hyperacusis (*sensitivity to light, sensitivity to noise)* tended to cluster together, with some interesting exceptions. For example, Patient 2’s network suggested that *nervousness* tended to co-occur with either *sadness* or *irritability*, but not both—rather, the presence of one appeared to preclude the presence of the other. In Patient 6’s network *sensitivity to light* and *sensitivity to noise* did not cluster together; the former was associated with *nausea* and *dizziness*, whereas the latter was associated with *memory problems* and *difficulty concentrating*.

There are currently no evidence-based treatments for persistent post-concussion symptoms. The diverse etiologies that underly symptomatology may contribute to this shortfall. Research suggests that psychosocial processes contribute to persistent symptoms, in at least a subset of those with post-concussion syndrome^8,21^. Other post-concussion disorders may arise from physiologic, vestibulo-ocular, or cervicogenic causes^22^. We speculate that distinct pathologies may correspond to symptoms’ temporal signatures and network topologies, and these differences may have implications for treatment and prognosis. Relating network features to concussion treatment response is purely speculative at this time. However, insights from network modeling have been applied to treatment of depression and anxiety^14,23^, and this work models a path forward towards personalized concussion care.

There are limitations to the present study that bear noting. The paths connecting symptoms within the network models reflect covariation among symptoms. Although we speculate about potential causal links, we cannot infer causality based on these results. Although the overall response rate to the EMA prompts was high (73%), 4 of 10 participants had response rates below 80%. Future research should design EMA protocols to maximize compliance, by making efforts to reduce the burden of the assessments and providing incentives for high compliance. The present study was powered for the person-specific data analysis, and as such, the total number of participants is small. Hence, the current study is underpowered to evaluate other questions of interest, such as associations between network density and cognitive outcomes or time to return to activities. These would be interesting topics for future research.

In conclusion, the present study is responsive to the growing consensus that personalized-care models are needed to effectively treat a variety of conditions. This paradigm shift in clinical approach calls for a corresponding shift in research methodology. We present EMA and person-specific analytic techniques as a way of characterizing idiosyncratic aspects of concussion symptoms. We believe these methods are particularly well-suited to studying heterogeneity in concussion symptom presentation and recovery course. Future research in larger samples should address how personalized dynamic symptom models may relate to patient characteristics, injury biomarkers, and treatment response.

## Supplementary information

Supplementary Information.
