# Supplementary material for: Person-Specific Methods for Characterizing the Course and Temporal Dynamics of Concussion Symptomatology: A Pilot Study
Source: Sci Rep. 2020 Jan 27;10:1248. doi: 10.1038/s41598-019-57220-1 (PMC6985195; doi:10.1038/s41598-019-57220-1)
Supplement: Supplementary file 1 — Supplementary Information. [file 41598_2019_57220_MOESM1_ESM.pdf]

Person-Specific Methods for Characterizing the Course and Temporal Dynamics of Concussion Symptomatology: A Pilot Study

Amanda R. Rabinowitz Ph.D.[1] & Aaron J. Fisher Ph.D.[2]

[1]Moss Rehabilitation Research Institute, Elkins Park, PA

[2]Department of Psychology, University of California Berkeley, Berkeley, CA

Corresponding Author: Amanda Rabinowitz, Ph.D.

Moss Rehabilitation Research Institute

50 Township Line Rd.

Elkins Park, PA 19027

Rabinowa@einstein.edu

Aaron J. Fisher, Ph.D.

Department of Psychology

University of California Berkeley

2121 Berkeley Way

Berkeley, CA 94720

afisher@berkeley.edu

Supplemental Table: Neuropsychological test results

ID	RVLT Immediate		RVLT Delay		TMT A		TMT B		WAIS PSI	
	acute	3 mon	acute	3 mon	acute	3 mon	acute	3 mon	acute	3 mon
**1**	1.75	2.08	1.04	1.44	**29**	**38**	42	51	105	120
**2**	0.41	1.62	1.50	2.00	42	56	62	49	111	120
**3**	2.33	2.89	1.00	2.18	66	80	66	48	120	127
**4**	-0.58	1.08	-0.16	0.64	**26**	51	43	53	89	114
**5**	1.04	2.52	1.00	1.00	42	62	56	66	89	92
**6**	-0.90	2.87	-0.17	2.00	47	74	61	79	**79**	102
**7**	0.58	2.25	0.24	1.44	43	65	51	46	102	114
**8**	1.28	1.19	0.70	0.7	**38**	42	54	59	100	108
**9**	**-1.56**	0.11	-0.76	-0.18	55	47	56	56	86	94
**10**	0.49	0.65	0.32	1.12	**38**	51	**24**	46	**84**	108

RVLT = Rey Auditory Learning Test, TMT = Trail Making Test, WAIS PSI = Wechsler Adult Intelligence Scale 4th Edition, Processing Speed Index. RVLT is in z-score units, Trails A and Trails B in T-score units, WAIS PSI in Standard Score units. Bold scores are 1 SD below the mean on demographically adjusted published norms.
